# Exploiting the Integrated Valorization of *Eucalyptus globulus* Leaves: Chemical Composition and Biological Potential of the Lipophilic Fraction before and after Hydrodistillation

**DOI:** 10.3390/ijms24076226

**Published:** 2023-03-25

**Authors:** Cátia. S. D. Oliveira, Patrícia Moreira, Maria T. Cruz, Cláudia M. F. Pereira, Artur M. S. Silva, Sónia A. O. Santos, Armando J. D. Silvestre

**Affiliations:** 1 CICECO—Aveiro Institute of Materials, Department of Chemistry, University of Aveiro, 3810-193 Aveiro, Portugal; 2 CNC—Center for Neuroscience and Cellular Biology, University of Coimbra, 3004-504 Coimbra, Portugal; 3 Faculty of Pharmacy, University of Coimbra, 3000-548 Coimbra, Portugal; 4 Faculty of Medicine, University of Coimbra, 3000-548 Coimbra, Portugal; 5 LAQV-REQUIMTE, Department of Chemistry, University of Aveiro, 3810-193 Aveiro, Portugal

**Keywords:** *Eucalyptus globulus* leaves, biorefinery, integrated exploitation, hydrodistillation, GC–MS analysis, cytotoxicity, triterpenic acids, ursolic acid

## Abstract

*E. globulus* leaves have been mainly exploited for essential oil recovery or for energy generation in industrial pulp mills, neglecting the abundance of valuable families of extractives, namely, triterpenic acids, that might open new ways for the integrated valorization of this biomass. Therefore, this study highlights the lipophilic characterization of *E. globulus* leaves before and after hydrodistillation, aiming at the integrated valorization of both essential oils and triterpenic acids. The lipophilic composition of *E. globulus* leaves after hydrodistillation is reported for the first time. Extracts were obtained by dichloromethane Soxhlet extraction and analyzed by gas chromatography-mass spectrometry. In addition, their cytotoxicity on different cell lines representative of the innate immune system, skin, liver, and intestine were evaluated. Triterpenic acids, such as betulonic, oleanolic, betulinic and ursolic acids, were found to be the main components of these lipophilic extracts, ranging from 30.63–37.14 g kg^−1^ of dry weight (dw), and representing 87.7–89.0% *w*/*w* of the total content of the identified compounds. In particular, ursolic acid was the major constituent of all extracts, representing 46.8–50.7% *w*/*w* of the total content of the identified compounds. Other constituents, such as fatty acids, long-chain aliphatic alcohols and *β*-sitosterol were also found in smaller amounts in the studied extracts. This study also demonstrates that the hydrodistillation process does not affect the recovery of compounds of greatest interest, namely, triterpenic acids. Therefore, the results establish that this biomass residue can be considered as a promising source of value-added bioactive compounds, opening new strategies for upgrading pulp industry residues within an integrated biorefinery context.

## 1. Introduction

In the last decades, the growing concern about the finitude of fossil resources, as well as the environmental impact resulting from their massive use, associated with greenhouse gas (GHG) emissions that result in climate change and global warming [1,2,3] have been pushing industry to develop fully sustainable processes and particularly those based on renewable raw materials. The exploitation of value-chains based on biomass has led to the emergence of the biorefinery concept, which still requires the development of efficient fractionation processes, allowing for the integrated exploitation of all biomass fractions and ensuring benefits to society and the environment, including their economic viability [3,4].

At the same time, in the last decades, the demand for new alternatives to petrochemical products has aroused great interest in the search for high-value compounds from renewable feedstocks, such as forest biomass. The forest activity, associated with the pulp and paper sector has a great impact on the world economy. In fact, Portugal is considered the 3rd largest European producer of pulp fibers [5].

**Figure 1:**
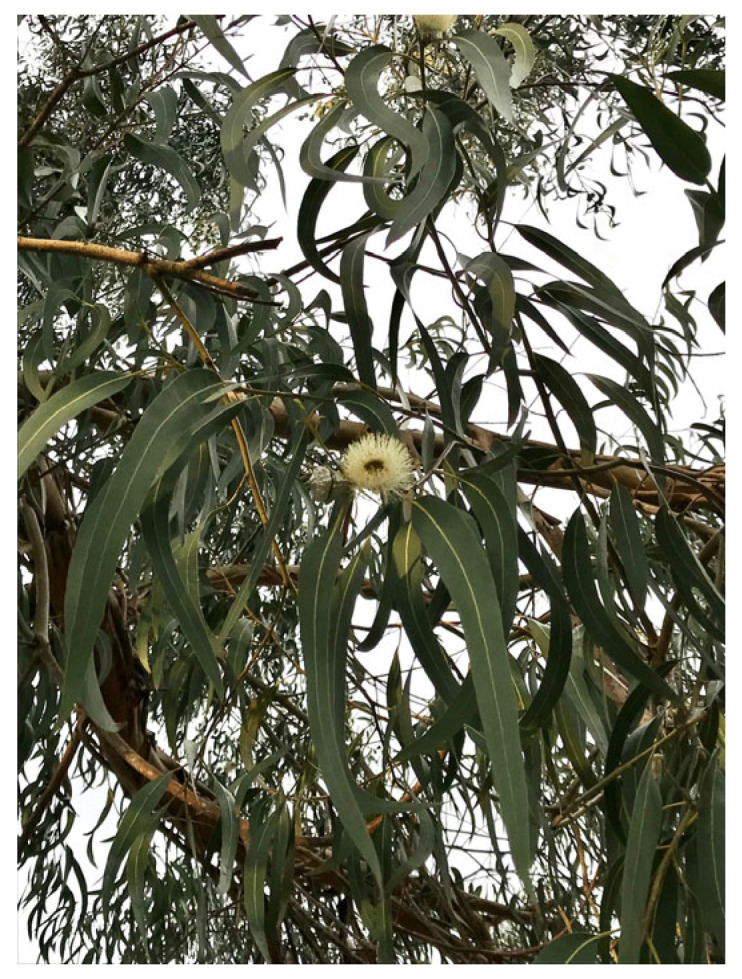
*Eucalyptus globulus* leaves.

*Eucalyptus globulus* is one of the most widely cultivated species in Portugal. According to the 6th National Forest Inventory (IFN6) published in 2019, eucalyptus is the forest species that occupies the largest planted area, approximately 844 kha, which corresponds to 26% of the Portuguese forest area [6]. The large-scale exploitation of *E. globulus* wood for pulp production generates large amounts of forest by-products, such as bark, branches and leaves (Figure 1) that have been widely used as solid fuel for power generation [4,7,8]. Nevertheless, the integrated valorization of these residues may represent a significant contribution to the paper and/or forestry sector’s profitability.

*E. globulus* pulping residues have been scrutinized as a source for added-value applications such as materials, chemicals or biofuels [9,10,11,12,13,14]. In the last years, particular interest has been devoted to the search for bioactive compounds from these resources. Some studies on *E. globulus* leaves report the presence of different families of compounds, namely, its essential oil (EO) [15,16,17], hydrophilic components, such as phenolic compounds [18,19,20,21], and lipophilic components, such as fatty acids, long-chain aliphatic alcohols, sterols, and triterpenic acids, such as betulinic, betulonic, oleanolic, and ursolic acids [12,22,23,24].

*Eucalyptus* leaf extracts and EO have long been used in the pharmaceutical, sanitary, agricultural, cosmetic, and food industries because of their beneficial and healthy properties [25,26]. In fact, traditionally, *Eucalyptus* leaves have been widely used for the treatment of various diseases such as influenza, dysentery, pulmonary tuberculosis, cystitis, diabetes, articular pain, fungal infections, dermatitis, scabies, and burns [25,27]. 

*E. globulus* is widely recognized and exploited as a source of EO that is composed mainly of monoterpenes and sesquiterpenes, which are obtained by hydrodistillation or steam distillation with extraction yields ranging from 1.2 to 2.7% (*w*/*w*) [15,16,17,28]. Nevertheless, after this process the leaf biomass remains underexploited, despite its richness in other valuable extractives fractions as, for example, triterpenic acids. The effect of the hydrodistillation process on *E. globulus* leaf extract composition is also unknown; therefore, this study aims to understand the effect of hydrodistillation on the composition of *E. globulus* leaf lipophilic extractives with emphasis on triterpenic acids, but including also the less abundant fractions of fatty acids and long-chain aliphatic alcohols in order to access the potential of the exploitation of this fraction integrated with EO.

In this vein, the lipophilic fraction of *E. globulus* leaves before and after hydrodistillation was obtained by Soxhlet extraction, and analyzed by gas chromatography-mass spectrometry (GC–MS) and their compositions were discussed in detail. In order to evaluate the safety of the different extracts to be exploited in different applications, their cytotoxicity was evaluated in cell lines representative of the innate immune system, skin, liver, and intestine, namely, on macrophages (RAW 264), fibroblasts (NIH/3T3), hepatocytes (HepG2) and colon cancer (Caco-2), respectively.

## 2. Results and Discussion

### 2.1. Extraction Yields

Lipophilic extracts of *E. globulus* leaves before and after hydrodistillation were obtained by Soxhlet extraction with dichloromethane (DCM). The extraction yields obtained were 19.5 ± 0.2% and 22.0 ± 0.6% of dw for *E. globulus* leaf DCM extracts before and after hydrodistillation, respectively. Although the values were approximate, the *E. globulus* leaves after hydrodistillation (EgLHD) showed a slightly higher extraction yield than before hydrodistillation (EgL), despite the fact that in this latter case EOs (1.7% of dw) were also extracted. These values were significantly higher than those previously reported for *E. globulus* leaves using non-polar organic solvents [22,29,30]. Rodrigues et al. [22] presented Soxhlet DCM extraction yields of 7.32% of dw and 2.38–2.89% of dw before and after a wax removal pretreatment, respectively. While other studies, also with *E. globulus* leaves, reported extraction yields of 2.2 and 9% of dw for solid–liquid extractions with hexane and DCM, respectively [24,30]. The lipophilic extractive yield of *Eucalyptus* leaves after hydrodistillation was only reported by El-Ghorab et al. [29] for a different species, namely, *E. camaldulensis,* with a value of 7.4 ± 0.6% of dw.

### 2.2. Chemical Characterization of the Lipophilic Extracts

**Figure 2:**
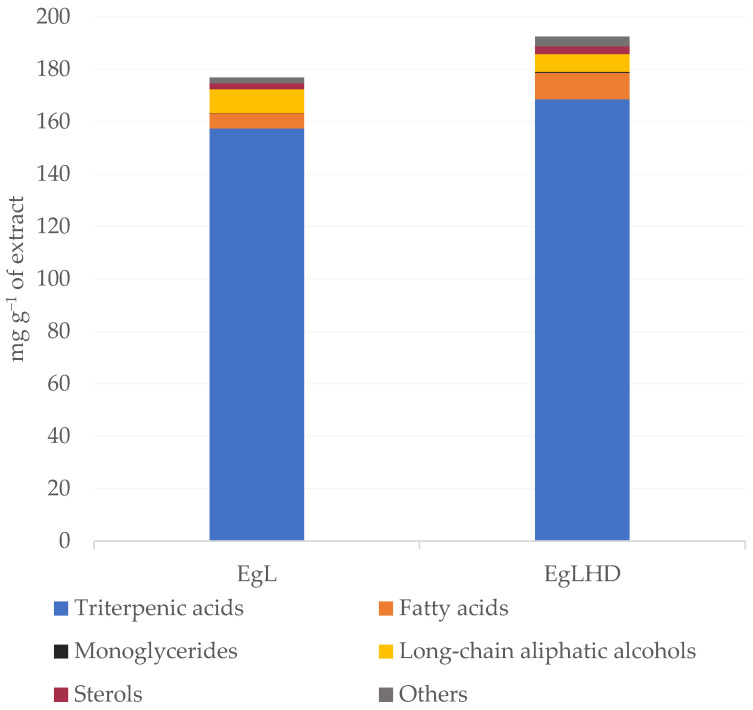
Main families of lipophilic compounds identified by GC–MS in dichloromethane (DCM) extracts of *E. globulus* leaves before (EgL) and after (EgLHD) hydrodistillation.

The chemical composition of DCM extracts from *E. globulus* leaves before and after hydrodistillation was studied in detail by GC–MS. The identification of the lipophilic components and the corresponding quantification in the studied extracts are summarized in Table 1 and Figure 2. This analysis excludes the EO components (mono and sesquiterpenic compounds) which have been the focus of another study [31].

To our knowledge, this is the first study reporting in detail the chemical characterization of the lipophilic fraction of *E. globulus* leaves before and after hydrodistillation.

In general, both extracts were mainly composed of triterpenic compounds, free fatty acids, and long-chain aliphatic alcohols. One sterol and one monoglyceride were also detected, among other minor components. The total contents of the identified compounds were 34.41 and 42.37 g kg^−1^ of dw in the EgL and EgLHD extracts, respectively. The higher contents of lipophilic compounds in the EgLHD extract may have been due to the disruption of the leaf’s cellular structures during hydrodistillation, that might have facilitated the extractability of these components.

#### 2.2.1. Triterpenic Compounds

Triterpenic compounds were the predominant lipophilic compounds in the *E. globulus* leaves extracts (Table 1) and accounted for nearly 87.7–89% of the total content of identified compounds, with the DCM extract from the EgLHD containing the highest amount with 37.14 g kg^−1^ of dw, and the DCM extract from the EgL with 30.63 g kg^−1^ of dw, respectively. Ursolic acid was the major triterpenic compound in both extracts, with contents of 17.46 g kg^−1^ of dw in the EgL and 19.82 g kg^−1^ of dw in the EgLHD. Previous studies on *E. globulus* leaves also identified ursolic acid as the majority triterpenic acid [12,22]. Considerable amounts of oleanolic acid were also detected in a range from 5.45 g kg^−1^ of dw in the EgL to 6.32 g kg^−1^ of dw in the EgLHD, followed by betulonic acid with 3.61 g kg^−1^ of dw in the EgL and 6.48 g kg^−1^ of dw in the EgLHD, and betulinic acid with 1.37 g kg^−1^ of dw in the EgL to 1.43 g kg^−1^ of dw in the EgLHD. According to the literature, triterpenic acids possess a wide range of biological activities. Ursolic and oleanolic acids have very low toxicity and are known for their significant antimicrobial, anti-inflammatory and antihyperlipidemic, antitumor, hepatoprotective, and cytotoxic activities, among others [32,33,34,35]. Betulonic acid has shown significant antiviral, antimalarial and anti-leishmanial activities and furthermore, cytotoxic properties against human cancer cell lines (e.g., HT29 colorectal carcinoma cells, KB oral epidermoid carcinoma and HONE-1 nasopharyngeal carcinoma) [36,37,38,39]. Whereas betulinic acid has been shown to exhibit a wide range of biological activities including anti-HIV, antimalarial, anti-inflammatory, antibacterial, anthelmintic and antioxidant properties [32,38,40,41]. This wide range of biological properties associated with the triterpenic acids identified in *E. globulus* leaves reveals the promising character of this biomass as a raw material for applications in the pharmaceutical, nutraceutical and cosmetic industries.

Additionally, acetyl derivatives of triterpenic acids (e.g., oleanolic, betulinic and ursolic acids) were identified in significant amounts in all the extracts. The most abundant acetylated compound was 3-acetylbetulinic acid, followed by 3-acetyloleanolic and 3-acetylursolic acids. All these triterpenic acids have already been reported as components of *E. globulus* [8,9,12,22,42].

#### 2.2.2. Fatty Acids

Fatty acids (C_14_ to C_30_) were detected in both extracts (Table 1), accounting for 3.3–5.3% of the total compounds identified. Total saturated fatty acids accounted for 1.13 and 2.11 g kg^−1^ of dw for the EgL and EgLHD, respectively. Hexadecanoic acid was the predominant saturated fatty acid found in the DCM extracts from the EgLHD, with a content of 0.58 g kg^−1^ of dw, while it only showed a content of 0.11 g kg^−1^ of dw in the extract of EgL. Hexadecanoic acid is one of the most common saturated fatty acids and also the most prevalent in body lipids [43]. This compound, both in acid and sodium salt form, is widely used in a variety of applications, such as food additives, cosmetic formulations, waterproofing materials, organic synthesis, etc. [44]. Triacontanoic and hexacosanoic acids were the most abundant saturated fatty acids for the DCM extract from the EgL, with contents of 0.39 and 0.33 g kg^−1^ of dw, respectively. In the extract from the EgLHD, the triacontanoic and hexacosanoic acids showed higher contents (i.e., 0.53 and 0.35 g kg^−1^ of dw, respectively). Fatty acids are the main constituents of the cell membrane structure with saturated fatty acids being essential for energy, cell membranes, hormone production, and organ cushioning [43]. 

Regarding the unsaturated fatty acids, the lipophilic extracts presented lower amounts than the saturated fatty acids. The extract from the EgLHD presented larger amounts of unsaturated fatty acids than before hydrodistillation, showing a content of 0.09 g kg^−1^ of dw, whereas only traces of unsaturated fatty acids were found in the DCM extract from the EgL. The most abundant unsaturated fatty acid was *cis*-octadec-9-enoic acid with a content of 0.07 g kg^−1^ of dw in the EgLHD.

#### 2.2.3. Monoglycerides and Sterols

The only monoglyceride detected, and in rather low amounts, was 1-monohexadecanoin, with the highest and lowest values recorded for the EgLHD (0.03 g kg^−1^ of dw) and EgL (0.01 g kg^−1^ of dw), respectively (Table 1).

*β*-Sitosterol was the only sterol identified in the studied *E. globulus* leaves, accounting for 0.64 g kg^−1^ of dw in the EgLHD and 0.45 g kg^−1^ of dw in the EgL (Table 1). *β*-Sitosterol had already been identified in the leaf waxes of *E. globulus*, and in other morphological parts such as bark, fruit, wood, etc. [8,9,12]. This phytosterol has a relevant added-value to the extracts, since it is reported to exhibit analgesic, anti-inflammatory, anti-proliferative, hypocholesterolemic, anti-cholesterolemic, anti-helmenthic, anti-diabetic, anti-atherogenic and antibacterial activities against *E. coli* and *Salmonella enterica Typhimurium*, among others [44,45]. *β*-Sitosterol, therefore, is already considered a functional bioactive compound, which can be used in nutraceutical and food products (called functional foods), and the conditions of use of its health claims have already been established in Commission Regulation (EU) No. 686/2014 [46].

#### 2.2.4. Long-Chain Aliphatic Alcohols

Four long-chain aliphatic alcohols (C_24_ to C_30_) were also detected in both *E. globulus* leaf extracts representing about 3.6–5.1% of the total lipophilic compounds identified (Table 1). The DCM extract from the EgL showed the highest amount with 1.76 g kg^−1^ of dw and the extract from the EgLHD showed the lowest amount (1.51 g kg^−1^ of dw). Triacontan-1-ol was the major constituent of this lipophilic class in the extracts with contents ranging from 0.86 to 1.01 g kg^−1^ of dw in the EgLHD and EgL, respectively, while tetracosan-1-ol was the minor aliphatic alcohol (with 0.04 g kg^−1^ of dw in the EgLHD and 0.06 g kg^−1^ of dw in the EgL).

#### 2.2.5. Other Compounds

Finally, other minor compounds were also present in the different extracts. A small amount of tyrosol and gallic acid was found in the EgLHD (Table 1), accounting for 0.01 g kg^−1^ of dw and 0.04 g kg^−1^ of dw, respectively. 

Glycerol was detected in an amount of 0.06 g kg^−1^ of dw in the EgLHD and traces in the EgL. *α*-Tocopherol and 1,6-dihydroxy-2-methylanthraquinone were identified in both extracts. In the case of *α*-tocopherol, also known as vitamin E, it is a lipophilic/liposoluble antioxidant, and reports indicate that it plays an important role in skin protection. Due to its properties, this compound is widely used as a low-cost antioxidant in cosmetic formulations and also as a food preservative [44,47].

### 2.3. Cell Viability of Lipophilic Extracts

**Figure 3:**
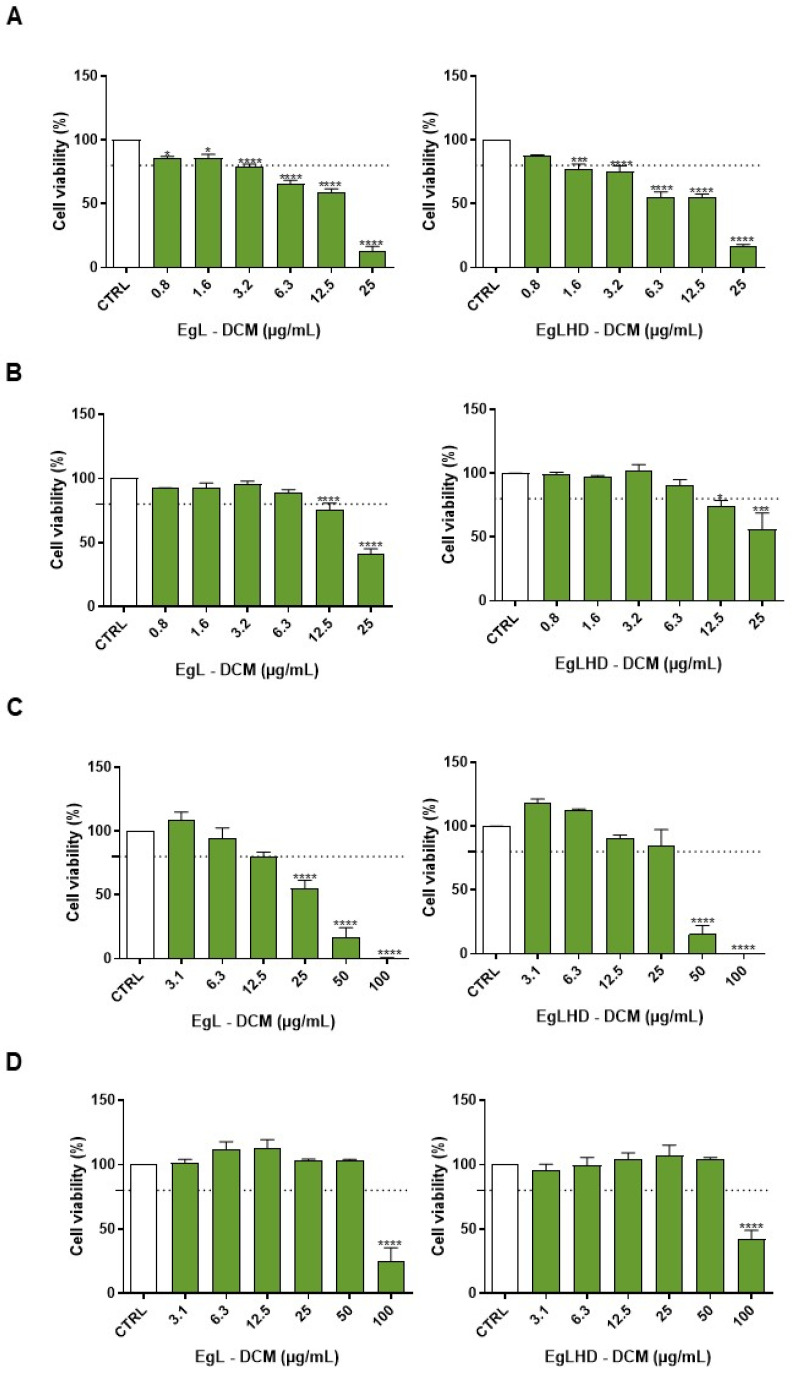
Effect of the lipophilic extracts from *E. globulus* leaves before (EgL) and after (EgLHD) hydrodistillation obtained with dichloromethane (DCM) on the cell viability of RAW 264.7 macrophages (**A**), NIH/3T3 fibroblasts (**B**), HepG2 hepatocytes (**C**), and Caco-2 intestinal (**D**) cells. The cells were treated for 24 h with 0–100 µg mL^−1^ of EgL or EgLHD, and the cell viability was evaluated using a 3-(4,5-dimethylthiazol-2-yl)-2,5-diphenyltetrazolium bromide (MTT) reduction assay. Cells treated with the medium alone were used as a control (CTRL). The results were expressed as the percentage (%) of cell viability relative to the CTRL and represent the mean ± standard error of the mean (SEM) of at least three independent experiments performed in triplicate. The statistical analysis was carried out by one-way analysis of variance (ANOVA) followed by a Dunnett’s multiple comparison test. * *p* < 0.05, *** *p* < 0.001, and **** *p* < 0.0001: significantly different compared to the CTRL.

The cytotoxicity of lipophilic extracts from the EgL and EgLHD obtained with DCM was evaluated, by the assessment of the cell viability using the MTT assay, in cell lines representative of the innate immune system, skin, liver, and intestine, namely, macrophages (RAW 264.7), fibroblasts (NIH/3T3), hepatocytes (HepG2), and intestinal cells (Caco-2) (Figure 3). Macrophages (Figure 3A) were the more sensitive cells to the toxicity of the extracts. After a 24 h treatment, the EgL and EgLHD extracts obtained with DCM were devoid of toxicity (i.e., they did not reach 20% of cellular mortality) at concentrations below 1.6 µg mL^−1^ and 0.8 µg mL^−1^, respectively. In fibroblasts (Figure 3B), an absence of toxicity was observed after a 24 h treatment with the lipophilic extracts at a concentration below 6.3 µg mL^−1^ for the EgL and EgLHD. Regarding hepatocytes (Figure 3C), non-toxic effects of the lipophilic extracts were observed at 24 h for concentrations below 12.5 µg mL^−1^ for the EgL, and 25 µg mL^−1^ for the EgLHD. In intestinal cells (Figure 3D), which were the cell lines more sensitive to the toxic effects of the extracts, an absence of toxicity was found after a 24 h incubation with both extracts at concentrations below 50 µg mL^−1^. In general, no differences in terms of toxicity were observed between the lipophilic extracts of *E. globulus* leaves before or after hydrodistillation. No studies in the literature report the toxicity of lipophilic extracts from eucalyptus; however, there are some studies in the same cells with ursolic acid, which was the major compound found in these extracts. For example, Yang et al. (2015) determined that around 30 µg mL^−1^ of ursolic acid causes 50% mortality to hepatocytes HepG2 after 24 h exposure [48], while other studies revealed that approximately 20–40 µg mL^−1^ of ursolic acid is non-toxic to the same cell line. Here, 25 µg mL^−1^ of the lipophilic extracts, which were composed of 40–44% ursolic acid, were safe to hepatocytes [49,50,51]. In addition, some studies have revealed that ursolic acid showed a low toxicity at approximately 15 µg mL^−1^ to RAW 264.7 macrophages after 24 h treatment [52], while almost 10 µg mL^−1^ was devoid of toxicity in peritoneal macrophages [53]. On the other hand, one study also with RAW 264.7 macrophages did not observe toxic effects in concentrations below 5 µg mL^−1^ after a 48 h incubation [54].

These studies revealed the susceptibility of macrophages to ursolic acid, which is in accordance with our study where macrophages were the cells more sensitive to the lipophilic extracts (at safe concentrations lower than 0.8–6.3 µg mL^−1^). Fibroblasts were the second cell line more sensitive to the lipophilic extracts, with safe concentrations between 6.3–12.5 µg mL^−1^. Actually, an in vitro study in the literature demonstrated that concentrations exceeding 10 µg mL^−1^ influenced HSF fibroblasts viability after a 24 h treatment [55]. Regarding intestinal cells, the more resistant cells to the toxicity of the lipophilic extracts in our study were with safe concentrations from 50 µg mL^−1^, but a study previously reported that concentrations above 10 µg mL^−1^ ursolic acid were toxic to intestinal Caco-2 cells [56]. This result is not similar to our study because we observed a low toxicity of the lipophilic extracts in intestinal cells; however, no exposure period of ursolic acid was mentioned in the previous study. A cytotoxicity screening of the lipophilic extracts obtained from *E. globulus* leaves before and after hydrodistillation was performed for the first time in cell lines representing the immune system, skin, liver, and intestine, disclosing its safe concentrations. The main compound identified in these extracts, namely, ursolic acid, possesses relevant biological effects, including anti-inflammatory, anticancer, antidiabetic, antioxidant, and antibacterial effects [57], and has been involved in a range of pharmacological applications, which are associated with the prevention of several diseases [58], such as skin conditions [59,60,61], liver, and intestinal damage [62,63,64], as well as inflammatory diseases, particularly in diabetes [65,66]. The bioactivities of the lipophilic extracts from *E. globulus* leaves, however, have not been studied yet; therefore, our study reveals the potential of lipophilic extracts from *E. globulus* leaves regarding their future incorporation in pharmaceutical formulations, and it supports the argument that their bioactivities should be further investigated.

## 3. Materials and Methods

### 3.1. Reagents

The dichloromethane (p.a., ≥99% purity) and ethanol (p.a., ≥99% purity) were supplied by Fisher Scientific (Thermo Fisher Scientific, Waltham, MA, USA). The pyridine (p.a., ≥99.5% purity), *N*,*O*-bis(trimethylsilyl)trifluroacetamide (99% purity), trimethylchlorosilane (99% purity), tetracosane (99% purity), hexadecanoic acid (≥99% purity), pentadecan-1-ol (99% purity), stigmasterol (95% purity), and ursolic acid (≥98% purity) were supplied by Sigma Chemical Co (Madrid, Spain). The gallic acid (≥97.5% purity) was purchased from Sigma-Aldrich (Merck, Darmstadt, Germany). The Dulbecco’s Modified Eagle’s Medium (DMEM), sodium bicarbonate, sodium pyruvate, non-essential amino acids, L-glutamine, glucose, phenol red, trypsin-ethylenediamine tetraacetic acid (EDTA) solution 1X and 3-(4,5-dimethylthiazol-2-yl)-2,5-diphenyltetrazolium bromide (MTT) were purchased from Sigma-Aldrich (St. Louis, MO, USA). The fetal bovine serum (FBS), and penicillin-streptomycin were obtained from Gibco (Carlsbad, CA, USA).

### 3.2. Samples Collection

*E. globulus* leaves, representative of harvesting biomass residues, were sampled from 6-year-old *E. globulus* trees, in October 2018, randomly selected from a property of “The Navigator Company”, Braçal (GPS coordinates 40°44′5.388 N, 8°23′53.97 W), in the region of Sever do Vouga, Portugal.

### 3.3. Hydrodistillation

Following the process of recovery of essential oils used at an industrial scale, a sample of fresh *E. globulus* leaves was subjected to a hydrodistillation process until the complete extraction of the EO (2–3 h), using the modified Clevenger apparatus [31]. The EO was obtained with a 1.7% yield. The main components of the EO were 1,8-cineole (72.3%), *a*-pinene (9.4%), *E*-pinocarveol (3.6%), limonene (2.3%), globulol (1.6), pinocarvone (1.4%) and *a*-terpinyl acetate (1.2%) [31].

Then, so that the water content did not compromise the extraction of the lipophilic contents, hydrodistilled leaves were air-dried until reaching a constant weight and grounded to obtain a biomass with a particle size less than 2/3 mm before extraction.

### 3.4. Preparation of Lipophilic Extracts

Samples of *E. globulus* leaves before and after hydrodistillation (ca. 12 and 10 g of dried biomass, respectively) were submitted, in triplicate, to Soxhlet extractions with DCM for a period of 8 h. The solvent was evaporated to dryness, at a low-pressure and 35 °C and the results were expressed as a percentage of dw. DCM is a very selective solvent to extract lipophilic compounds from biomass [10].

### 3.5. GC–MS Analysis

Before the GC–MS analysis, aliquots containing about 15 to 20 mg of each dried extract were dissolved in 250 μL of pyridine containing 0.4 mg of tetracosane (internal standard) and then, 250 μL of *N*,*O*-bis(trimethylsilyl)trifluoroacetamide and 50  μL of trimethylchlorosilane were added to converting compounds with hydroxyl and carboxyl groups into trimethylsilyl ethers and esters, respectively. The mixture remained at 70 °C for 30 min and the derivatized extracts were analyzed by GC–MS [42,67].

GC–MS analysis were carried out in a GC–MS-QP2010 Ultra (Shimadzu, Kyoto, Japan). The compounds were separated in a DB-1 J&W capillary column (with a 30 m × 0.32 mm inner diameter, and 0.25 µm film thickness), using helium as the carrier gas (40 cm s^−1^). The chromatographic conditions were as follows: initial temperature, 80 °C for 5 min; temperature rate, 4 °C min^−1^ up to 260 °C, 2 °C min^−1^ up to 285 °C, which was maintained for 15 min. The injector temperature was 250 °C, and the transfer-line temperature was 290 °C, while the split ratio was 1:50. The mass spectrometer was operated in the electron impact mode with an energy of 70 eV, and the data were collected at a rate of 1 scan s^−1^ over a range of m/z 35–900. The ion source was kept at 250 °C [68].

The eluted compounds identification was made by comparing their mass spectra (MS) with the equipment’s mass spectral library (NIST Mass Spectral Library), by comparing the MS fragmentation profiles with data from the literature [12,19,22] and by the co-injection of standards.

For the semi-quantitative analysis, the GC–MS apparatus was calibrated with pure reference standards representative of the main families of compounds present in the lipophilic extracts, namely, hexadecanoic acid, pentadecan-1-ol, stigmasterol, ursolic acid and gallic acid, in relation to tetracosane (the internal standard), which allowed to determine the respective response factors. The compounds were quantified by their peak areas in relation to tetracosane (the internal standard), corrected using the response factors, and their abundance expressed in mg g^−1^ of extract and g kg^−1^ of dw of biomass.

Each of the three extracts, prepared from leaves before and after hydrodistillation, was analyzed in duplicate (*n* = 6). The results presented are the average of the concordant values obtained (with less than a 5% variation between aliquots of the same sample and between tripled extracts of the same type of extraction).

### 3.6. Cell Culture

The mouse fibroblasts (NIH/3T3, ATCC CRL-1658, Manassas, VA, USA), and human colorectal adenocarcinoma (Caco-2, ATCC HTB-37, Manassas, VA, USA) cell lines were cultured with DMEM (#D5648), supplemented with 10% (*v*/*v*) heat-inactivated FBS, 1% (*v*/*v*) antibiotic solution (from a 10,000 U mL^−1^ penicillin, and 10 000 µg mL^−1^ streptomycin stock), 3.7 g L^−1^ of sodium bicarbonate and 1 mM sodium pyruvate. The culture medium of the Caco-2 cell line was additionally supplemented with 1% (*v*/*v*) non-essential amino acids. The mouse leukaemic macrophages cell line (RAW 264.7, ATCC TIB-71, Manassas, VA, USA) was cultured in DMEM (#D5648) supplemented with 10% (*v*/*v*) non-inactivated FBS, a 1% (*v*/*v*) penicillin/streptomycin antibiotic solution, 1.5 g L^−1^ of sodium bicarbonate, and 1 mM sodium pyruvate. The human liver hepatocellular (HepG2, ATCC HB-8065, Manassas, VA, USA) cell line was cultured in DMEM (#D5030) supplemented with 10% (*v*/*v*) heat-inactivated FBS, a 1% (*v*/*v*) penicillin/streptomycin antibiotic solution, 1.5 g L^−1^ of sodium bicarbonate, 1 mM sodium pyruvate, 4 mM L-glutamine, 1 g L^−1^ of glucose and phenol red. The cells were cultivated in 75 cm^2^ flasks in a humidified 5% CO_2_-95% air atmosphere at 37 °C, and the medium was changed every 2–3 days. For passage and sub-culturing, the fibroblasts, hepatocytes, and intestinal cells were detached using a trypsin-EDTA solution 1X when the cells reached a 70–80% confluence, while the macrophages were detached with a cell scrape. The cells were sub-cultured over a maximum of ten passages [31].

### 3.7. Cell Viability Evaluation

For the assessment of cell viability, the MTT reduction assay was performed. RAW 264.7, Caco-2, HepG2, and NIH/3T3 cells were seeded in 96-well plates at a density of 9.6, 5, 2.5 or 1 × 10^4^ cells/well, respectively, and allowed to stabilize for 24 h. The next day, the culture medium was removed and substituted by an exposure medium (i.e., DMEM supplemented with 1% (*v*/*v*) FBS). The cells were incubated for 24 h at 37 °C with 0–100 µg mL^−1^ of lipophilic extracts from *E. globulus* leaves before and after hydrodistillation obtained with DCM. The extracts were added from stock solutions prepared in DMSO and stored at −20 °C. Cells treated with the medium alone were used as a control. After the incubation period, the medium was aspirated after the incubation period and a solution of 0.5 mg mL^−1^ MTT prepared in Krebs medium (i.e., 140 mM NaCl, 5 mM KCl, 1 mM NaH_2_PO_4_, 1 mM MgCl_2_, 9.6 mM Glucose, 20 mM HEPES, 1.5 mM CaCl_2_, and a pH of 7.4) was added. The cells were incubated with MTT at 37 °C for 30 min (RAW 264. 7 cells), 1 h (HepG2 cells), 2 h (Caco-2 cells) or 4 h (NIH/3T3 cells). After that, the MTT solution was aspirated and DMSO was added to dissolve the formed formazan crystals. The absorbance was measured after 10 min of shaking, at 570 nm using a SpectraMax Plus 384 Spectrophotometer (Molecular Devices, San Jose, CA, USA). The results of at least three independent experiments made in triplicate were expressed as a percentage (%) of the absorbance value obtained in the control, which was considered 100%, and were graphically presented as a % of the cell viability versus the concentration of the extracts [69].

#### Statistical Analysis

The results are represented as the mean ± standard error of the mean (SEM) of the indicated number of experiments. The normality of the data distribution was evaluated using the D’Agostino and Pearson and Shapiro–Wilk normality tests. Statistical comparisons between the groups were performed by a one-way analysis of variance (ANOVA) followed by a Dunnett’s multiple comparison test. Significance was accepted at *p* values < 0.05. The GraphPad Prism software (8.0.2, GraphPad Software Inc., San Diego, CA, USA) was used to perform the statistical analysis.

## 4. Conclusions

The present study highlights promising insights into the chemical composition and cytotoxicity of lipophilic extracts of *E. globulus* leaves before and after hydrodistillation. To our knowledge, this is the first study of the chemical composition of hydrodistilled leaves. The obtained extracts were characterized in detail by GC–MS, allowing the identification and quantification of 31 compounds, including different families of compounds, such as triterpenic compounds, fatty acids, long-chain aliphatic alcohol, only one sterol, *β*-sitosterol, and other minor compounds. DCM extracts of the leaves before and after hydrodistillation were shown to exhibit valuable bioactive compounds, namely, triterpenic acids (e.g., betulonic, oleanolic, betulinic and ursolic acids) that are associated with numerous biological activities. The majority compound was ursolic acid, with contents ranging from 17.46–19.82 g kg^−1^ of dw. Interestingly, the extracts of the hydrodistilled leaves showed a higher content of the identified compounds than the non-hydrodistilled leaves.

The non-toxic concentrations of the extracts in intestinal cells, hepatocytes (liver), fibroblasts (skin) and macrophages (innate immune system) were determined. No significant differences in toxicity were observed between the extracts obtained from the leaves before or after hydrodistillation. Macrophages were shown to be the most sensitive cells to the extracts (with safe concentrations less than or equal to 0.8 µg mL^−1^) and intestinal cells the most resistant (with non-toxic concentrations less than or equal to 50 µg mL^−1^).

This study highlights the potential of *E. globulus* leaves, promoting their economic exploitation as a source of bioactive compounds with potential applications in pharmaceutical, nutraceutical and cosmetic formulations, which can only be implemented after the development of sustainable extraction methodologies, as well as a thorough technical–economic evaluation, and finally, an analysis to ensure the ecological impact of its exploitation. Finally, this study indicates that an integrated and sustainable exploitation of the species *E. globulus* can be considered, combining the exploitation of the leaves, for the recovery of EO and extracts enriched in value-added compounds, along with the exploitation of the wood, which is the main raw material for pulp production.

## Figures and Tables

**Table 1:** Compounds identified in dichloromethane (DCM) extracts of *E. globulus* leaves before (EgL) and after (EgLHD) hydrodistillation expressed in mg g^−1^ of extract and g kg^−1^ of dw biomass.

Rt_(min)_	Compound	mg g^−1^ of Extract		g kg^−1^ of dw	
		EgL	EgLHD	EgL	EgLHD
	Triterpenic acids	157.42	168.53	30.63	37.14
68.11	Betulonic acid	18.56	29.40	3.61	6.48
68.93	Oleanolic acid	28.02	28.70	5.45	6.32
69.52	Betulinic acid	7.05	6.49	1.37	1.43
70.46	Ursolic acid	89.73	89.93	17.46	19.82
73.09	3-Acetyloleanolic acid	5.11	4.56	0.99	1.00
76.20	3-Acetylbetulinic acid	6.85	7.14	1.33	1.57
77.25	3-Acetylursolic acid	2.09	2.32	0.41	0.51
	Fatty acids	5.79	10.14	1.13	2.23
	*Saturated fatty acids*	*5.79*	*9.57*	*1.13*	*2.11*
30.83	Tetradecanoic acid	0.29	0.52	0.06	0.11
35.74	Hexadecanoic acid	0.59	2.61	0.11	0.58
38.03	Heptadecanoic acid	tr	0.12	tr	0.03
40.24	Octadecanoic acid	0.13	0.26	0.03	0.06
48.26	Docosanoic acid	0.21	0.29	0.04	0.06
51.94	Tetracosanoic acid	0.33	0.47	0.06	0.10
55.97	Hexacosanoic acid	1.68	1.61	0.33	0.35
60.37	Octacosanoic acid	0.54	1.29	0.11	0.28
65.28	Triacontanoic acid	2.02	2.41	0.39	0.53
	*Unsaturated fatty acids*	*tr*	*0.42*	*tr*	*0.09*
39.32	Octadeca-9,12-dienoic acid	tr	tr	tr	tr
39.51	*cis*-Octadec-9-enoic acid	tr	0.34	tr	0.07
39.65	*trans*-Octadec-9-enoic acid	tr	0.08	tr	0.02
	*Diacids*	*tr*	*0.15*	*tr*	*0.03*
29.37	Nonanedioic acid	tr	0.15	tr	0.03
	**Long-chain aliphatic alcohols**	**9.06**	**6.85**	**1.76**	**1.51**
50.41	Tetracosan-1-ol	0.32	0.19	0.06	0.04
54.24	Hexacosan-1-ol	1.23	0.96	0.24	0.21
58.49	Octacosan-1-ol	2.33	1.79	0.45	0.39
63.06	Triacontan-1-ol	5.18	3.91	1.01	0.86
	**Monoglycerides**	**0.07**	**0.29**	**0.01**	**0.03**
47.57	1-Monohexadecanoin	0.07	0.29	0.01	0.03
	**Sterols**	**2.33**	**2.91**	**0.45**	**0.64**
62.61	*β*-Sitosterol	2.33	2.91	0.45	0.64
	**Others**	**2.13**	**3.70**	**0.42**	**0.82**
14.14	Glycerol	tr	0.28	tr	0.06
22.95	Tyrosol	n.d.	0.05	n.d.	0.01
34.03	Gallic acid	n.d.	0.19	n.d.	0.04
57.23	1,6-Dihydroxy-2-methylanthraquinone	1.16	0.99	0.23	0.22
58.03	*α*-Tocopherol	0.97	2.19	0.19	0.48
	**TOTAL**	**176.81**	**192.44**	**34.41**	**42.37**

Abbreviations: n.d.: not detected; Rt: retention time; tr: traces.
